# Compact Retention and Lineage-Specific Sequence Divergence of ALMT Genes in Acidophilic Vaccinium

**DOI:** 10.3390/plants15132086

**Published:** 2026-07-04

**Authors:** Bin Li, Wenhan Cheng, Xianyang Zhao, Rui Chen, Ruiyi Fan

**Affiliations:** 1 College of Food and Biology, Jingchu University of Technology, Jingmen 448000, China; libin0118@outlook.com (B.L.); kaven_53@163.com (W.C.); xyzhao2018@163.com (X.Z.); chenrui@jcut.edu.cn (R.C.); 2 Hubei Engineering Research Center for Specialty Flowers Biological Breeding, Jingmen 448000, China

**Keywords:** *Vaccinium*, ALMT, aluminum tolerance, comparative genomics, positive selection, gene family compactness, gene family evolution

## Abstract

Aluminum-activated malate transporter (ALMT) channels mediate root malate efflux, a key response for plant survival in acidic, aluminum-toxic soils. The acidophilic genus *Vaccinium* is horticulturally important, yet its ALMT family has remained uncharacterized. Leveraging two newly available chromosome-level genomes (*Vaccinium darrowii* and *Vaccinium duclouxii*), we present the first genus-wide characterization of this family. Across 11 angiosperms we identified 145 non-redundant ALMT loci, partitioned into six major subfamilies. MCScanX synteny revealed compact retention rather than expansion: diploid *Vaccinium* genomes encode only 8–10 ALMTs each, with at most one intra-species syntenic paralog pair, versus multiple whole-genome-duplication-derived pairs in apple (*Malus domestica*, 24 loci). Pairwise *Ka/Ks* was elevated within *Vaccinium* in three subfamilies, and codon-based PAML branch-site tests detected Bonferroni-significant positive selection on terminal *Vaccinium* branches in Subfamilies 5 and 4 (2ΔlnL = 40.65 and 12.95), yielding three Bayes Empirical Bayes candidate sites in Subfamily 5 (F249, E410, L411) and two in Subfamily 4 (E249, G399); Subfamily 2 was non-significant and is interpreted as relaxed constraint. All candidate residues map to C-terminal cytoplasmic regulatory regions rather than the transmembrane pore. These findings indicate that the compact *Vaccinium* ALMT repertoire retained its ancestral channel architecture while accumulating lineage-associated divergence in cytoplasmic regulatory regions; the identified residues are candidates for downstream functional validation rather than demonstrated drivers of acid-soil adaptation.

## 1. Introduction

Plants colonizing acidic soils face a complex suite of physicochemical challenges that include elevated mobilization of phytotoxic Al^3+^ from soil aluminosilicates, reduced availability of phosphate and several divalent cations, and a low rhizosphere pH that constrains nutrient uptake. A central component of the molecular response to these stresses is the secretion of low-molecular-weight organic acids, primarily malate and citrate, from the root apex into the rhizosphere, where they chelate Al^3+^ and mobilize occluded phosphate [1]. The aluminum-activated malate transporter (ALMT) family, first identified by the cloning of *TaALMT1* in wheat (*Triticum aestivum* L.) [2] and *AtALMT1* in *Arabidopsis thaliana* [3], encodes plasma-membrane anion channels that mediate malate efflux at the root apex and have been repeatedly implicated in genotype-level variation in plant Al tolerance and acid-soil performance across diverse angiosperm lineages.

The ALMT family has been characterized in genome-wide form in several model species. In A. thaliana, the family comprises approximately 14 canonical AtALMT members spanning multiple subcellular destinations and physiological roles [3]; our unified HMM filter (Section 2.2) retained 13 of these Arabidopsis loci (Table S2). In Malus domestica, the larger ALMT family (25 entries originally reported by Ma et al. [4]; 24 locus-level loci recovered in this work from the T2T GDT2T_hap1 assembly [5] after collapsing alternative transcripts to unique loci) reflects the Rosaceae-specific whole-genome duplication that occurred ~30–45 Mya [6], and several MdALMTs have been linked to fruit malate accumulation and acidity quantitative trait loci. Recent cryo-EM structures of Arabidopsis ALMT1 [7] and Glycine max ALMT12 [8], together with electrophysiological dissection of Arabidopsis QUAC1/ALMT12 [9] and Brachypodium distachyon ALMT12 [10], have established that ALMT channels function as homodimers with a transmembrane pore-forming module and a large cytoplasmic C-terminal domain (CHD) that mediates ligand-dependent gating, calmodulin binding, and voltage sensing. Recently, family-level characterization has also been reported in *Camellia sinensis* [11], where CsALMT6 mediates fluoride resistance, underscoring the lineage-specific functional diversification of ALMT paralogs across acid-soil-tolerant species.

Despite these advances, the *ALMT* family has not been examined in *Vaccinium*, a genus of approximately 450 species within Ericaceae that is exceptional among horticultural crops in its obligate acid-soil requirement. Blueberry, cranberry, and related *Vaccinium* species grow normally only in soils of pH 4–5.5 and develop chlorosis and stunted growth at neutral pH. This requirement is widely interpreted as a manifestation of co-evolution between *Vaccinium* hosts and their ericoid mycorrhizal symbionts [12], which themselves require acidic conditions, and is biochemically associated with reduced Fe acquisition and ammonium preference at neutral pH. The recent availability of high-quality reference genomes for five *Vaccinium* species—*Vaccinium corymbosum* cv. Draper [13], *Vaccinium macrocarpon* [14], *Vaccinium microcarpum* [14], *Vaccinium darrowii* [15], and the T2T gap-free *Vaccinium duclouxii* [16]—together with chromosome-scale assemblies of related Ericaceae and outgroup taxa, now permits the first comparative-genomic analysis of any organic-acid efflux gene family in this lineage.

In this study, we surveyed the ALMT gene family across 11 angiosperm species spanning Ericaceae, Theaceae, Actinidiaceae, Rosaceae, Vitaceae, and Brassicaceae, and characterized its evolution along the following four complementary axes: (i) family-size variation across the 11 species, with explicit comparison of Vaccinium against the Rosaceae reference *M. domestica;* (ii) orthogroup-level subfamily structure inferred by OrthoFinder, calibrated against a maximum-likelihood phylogeny of all 145 locus-level ALMTs; (iii) per-subfamily selective regime, assessed by pairwise Ka/Ks analysis and by codon-based branch-site tests of positive selection on terminal Vaccinium branches (PAML) [17]; and (iv) genomic mode of family-size evolution, dissected by using MCScanX (https://github.com/wyp1125/MCScanX; accessed on 20 April 2026) for synteny analysis and gene-duplication classification of the three chromosome-level genomes in the dataset (*M. domestica*, *Vaccinium duclouxii*, and *Vaccinium darrowii*). We frame three working hypotheses that motivate this analytical design. H1: Vaccinium species harbour an expanded ALMT family relative to non-acidophilic relatives, reflecting selection on organic-acid efflux capacity. H2: Vaccinium species harbour a compact ALMT family of largely ancestral composition combined with lineage-specific sequence divergence, particularly at sites involved in channel regulation. H3: ALMT family-size variation across angiosperms is dominated by lineage-specific whole-genome-duplication history and is independent of edaphic preference. The data presented below most strongly support H2, with the Vaccinium ALMT family showing a pattern of compact retention combined with subfamily-specific elevation of nonsynonymous substitution rates and discrete site-specific positive selection localized to predicted regulatory regions of two ALMT subfamilies.

## 2. Materials and Methods

### 2.1. Plant Materials and Genome Data

Eleven genomes representing diverse phylogenetic positions and edaphic preferences were selected for comparative analysis (Supplementary Table S1). These included the following five *Vaccinium* species spanning the acid-tolerant Ericaceae crown: *Vaccinium darrowii* (Vda; diploid) [15], *Vaccinium macrocarpon* “Stevens” (Vma; diploid cranberry) [14], *Vaccinium microcarpum* (Vmi; diploid small cranberry) [14], *Vaccinium duclouxii* (Vdu; diploid T2T assembly) [16], and *Vaccinium corymbosum* “Draper” (Vc; tetraploid highbush blueberry) [13]. *Rhododendron simsii* (Rs) was also included as a sister Ericaceae outgroup. Furthermore, two additional acid-tolerant species (*Camellia sinensis*, Cs, Theaceae; *Actinidia eriantha*, Ae, Actinidiaceae) and three reference species (*Malus domestica*, Md, Rosaceae; *Vitis vinifera*, Vv, Vitaceae; *Arabidopsis thaliana*, At) provided comparative context. Genome assemblies and gene annotations were retrieved from NCBI RefSeq (https://www.ncbi.nlm.nih.gov/refseq/; accessed on 15 April 2026), the Genome Database for Vaccinium (GDV; https://www.vaccinium.org/; accessed on 15 April 2026), and the NGDC Genome Warehouse (GWH; https://ngdc.cncb.ac.cn/gwh; accessed on 15 April 2026), and were subsequently standardized for downstream analysis. Detailed accession numbers, assembly versions, and protein/gene counts are provided in Supplementary Table S1.

### 2.2. Identification of ALMT Genes

The ALMT-family hidden Markov model (Pfam accession PF11744, version 35.0) was downloaded from InterPro (https://www.ebi.ac.uk/interpro/entry/pfam/PF11744; accessed on 20 April 2026). To capture functionally validated ALMT diversity, the following dual-query strategy was used: (i) HMMSEARCH (HMMER v3.3.2) [18] of PF11744 against the *A. thaliana* proteome (*E* < 1 × 10−5, score ≥ 50) identified 20 high-confidence Arabidopsis ALMT candidates; (ii) the same procedure on *M. domestica* GDT2T_hap1 [5] identified 35 high-confidence Malus ALMT candidates. These two seed sets pool transcript- and isoform-level HMM hits and are used here purely as BLASTP (BLAST+ v2.13) query libraries; the corresponding locus-level counts retained in the final dataset (13 At loci and 24 Md loci) emerge only after the full downstream pipeline (per-species CD-HIT v4.8.1, length and HMM-coverage thresholds, and locus-level transcript deduplication) and are reported in Table 1. The combined 55 ALMT protein sequences were used as BLASTP queries (BLAST+ v2.13, *E* < 1 × 10−10, identity > 30%, query coverage > 50%) against each of the 11 proteomes.

Putative ALMT genes were defined as the union of HMMSEARCH hits (score > 30; deliberately relaxed at this union step to capture divergent ALMT-like sequences, then re-tightened to bit-score ≥ 50 in the validation step below) and BLASTP hits passing the above thresholds per species. Per-species redundancy was reduced by CD-HIT (v4.8.1) [19] at 95% identity. Sequences shorter than 200 amino acids were excluded. Surviving candidates were re-validated by HMMSEARCH against PF11744, retaining only those with bit score ≥ 50 and envelope coverage ≥ 60% of the ALMT domain. Finally, alternative transcripts mapping to the same Parent gene (per the species GFF3) were collapsed to a single representative isoform per locus. The final set comprised 145 non-redundant locus-level ALMT loci across 11 species (Supplementary Tables S2 and S3; per-species count in Table 1).

**Table 1:** ALMT gene family size across the 11 angiosperm species analyzed in this study. Species are ordered by phylogenetic position (cf. Figure 1); edaphic preference and ploidy are compiled in Supplementary Table S1.

Species	Family	Edaphic Preference	Ploidy	ALMT Genes (*n*)
*Vitis vinifera*	Vitaceae	Neutral to alkaline	2×	12
*Arabidopsis thaliana*	Brassicaceae	Neutral	2×	13
*Malus domestica*	Rosaceae	Slightly acidic to neutral	2×	24
*Camellia sinensis*	Theaceae	Acidic (pH 4.5–6)	2×	15
*Actinidia eriantha*	Actinidiaceae	Acid-tolerant (slightly acidic)	2×	21
*Rhododendron simsii*	Ericaceae	Acidic (pH 4–6)	2×	11
*Vaccinium macrocarpon*	Ericaceae	Acidic (pH 4–5)	2×	10
*Vaccinium microcarpum*	Ericaceae	Acidic (pH 4–5.5)	2×	9
*Vaccinium corymbosum*	Ericaceae	Acidic (pH 4.5–5.5)	4×	13
*Vaccinium darrowii*	Ericaceae	Acidic (pH 4–5.5)	2×	8
*Vaccinium duclouxii*	Ericaceae	Acidic (pH 4–5.5)	2×	9

**Figure 1:**
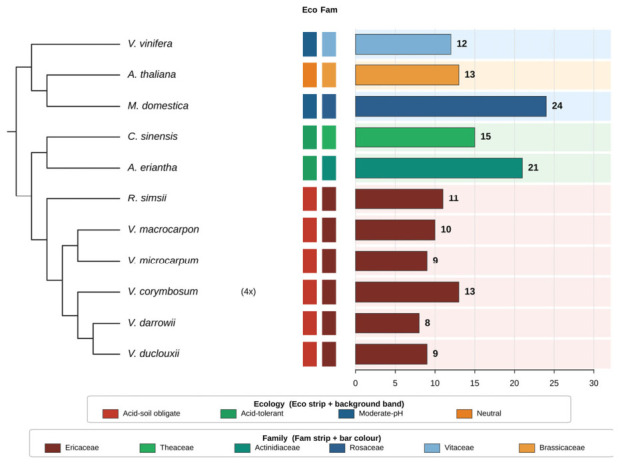
Genome-wide ALMT gene family size across 11 angiosperm species. ALMT protein count per species is shown as a horizontal bar chart, with species ordered by APG IV phylogenetic position (tree shown on the left). Each species is annotated for its edaphic preference (Eco strip) and phylogenetic family (Fam strip). Bar background colour denotes edaphic preference. Vaccinium species (five acid-soil obligate species) carry compact families of 8–10 members per diploid genome; the tetraploid *V. corymbosum* carries 13 members. The Rosaceae reference *M. domestica* (Rosaceae) carries the largest family (24 locus-level members), reflecting the recent Rosaceae-specific whole-genome duplication.

### 2.3. Phylogenetic Analysis

The 145 locus-level ALMT protein sequences were aligned using MAFFT v7.526 with the L-INS-i algorithm (-localpair-maxiterate 1000) [20]. Poorly aligned columns were removed with trimAl v1.4.rev22 (-automated1) [21], yielding the final trimmed alignment of 326 columns retained from the initial MAFFT L-INS-i alignment. The ALMT family is divergent in its non-transmembrane regions and shows substantial length variation between subfamilies; the -automated1 heuristic therefore retained the conserved, reliably aligned columns shared across the 145 sequences. Phylogenetic inference was performed on this conserved core, and the resulting topology recovered the six major OG-defined subfamilies with high ultrafast-bootstrap support (≥70 in the majority of internal nodes). Maximum-likelihood phylogenetic inference was performed with IQ-TREE 2.2.2.2 [22] using ModelFinder as implemented in IQ-TREE v2.2.2.2 for automatic model selection (-m MFP) under the Bayesian Information Criterion (BIC), which penalizes overfitting by additional parameters and is therefore preferred for divergent multi-species gene families; JTT + F + R5 was selected as the best-fit model. Branch support was assessed by 1000 ultrafast bootstrap replicates (-bb 1000) [23] and 1000 SH-aLRT replicates (-alrt 1000). The consensus tree (log-likelihood = −25,667.56) was midpoint-rooted—chosen over outgroup rooting because no non-ALMT outgroup provides comparable taxonomic coverage across all 145 leaves of the alignment—and visualized in iTOL v6 [24].

### 2.4. Orthogroup Inference and Subfamily Classification

Whole-proteome orthology relationships across the 11 species were inferred using OrthoFinder v3.1.4 [25] with DIAMOND v2 [26] for all-versus-all protein similarity, the -M msa-T iqtree3 option for gene tree-based orthogroup refinement, FAMSA (https://github.com/refresh-bio/FAMSA; accessed on 20 April 2026) for alignment, and MCL clustering as implemented in OrthoFinder at inflation parameter 1.2. All 145 retained ALMT proteins were mapped to OrthoFinder orthogroups based on protein ID matching against the Orthogroups.txt output. ALMTs were assigned to nine distinct orthogroups, with six major OGs each containing ≥12 ALMTs (OG0001431, OG0002701, OG0001688, OG0006223, OG0008930, and OG0012384). These six major OGs collectively contain 142 of the 145 ALMT loci (98%) and are treated as the major ALMT subfamilies in downstream analyses. Singleton orthogroups (≤2 members) were retained for completeness but excluded from per-OG Ka/Ks comparison.

For downstream analyses, we ranked the six largest orthogroups by total member count in descending order and designated them Subfamily 1 (OG0001431, *n* = 38), Subfamily 2 (OG0002701, *n* = 31), Subfamily 3 (OG0001688, *n* = 26), Subfamily 4 (OG0006223, *n* = 19), Subfamily 5 (OG0008930, *n* = 16), and Subfamily 6 (OG0012384, *n* = 12); three remaining singletons are reported as “minor” orthogroups (see Supplementary Table S2).

### 2.5. Ka/Ks Analysis

Coding sequences (CDSs) for each ALMT gene were extracted from species genomes using gffread v0.12.7 [27] on cleaned GFF3 files. Protein-to-mRNA-to-CDS ID resolution was performed per species reflecting its annotation source format (NCBI: protein_id → Parent = rna-linkage; GDV: shared mRNA ID; NGDC: OriID = mapping in protein FASTA headers). Per-OG codon alignments were generated by (i) aligning ALMT protein sequences with MAFFT v7.526 (-auto), (ii) back-translating to codon alignment by mapping codons from the original CDS onto the protein alignment in three-nucleotide blocks. Pairwise non-synonymous (*Ka*) and synonymous (*Ks*) substitution rates were computed with KaKs_Calculator v2.0 [28] using the Nei–Gojobori (NG) method.

For each of the six major OGs, pairwise *Ka/Ks* ratios were partitioned into the following three classes: Vac–Vac (both members from any *Vaccinium* species), Out–Out (neither member from *Vaccinium*), and Vac–Out (one member from each). The fold-change in Vac–Vac vs. Out–Out *Ka/Ks* was reported per OG (Table 2).

### 2.6. Branch-Site Test of Positive Selection

To detect site-specific positive selection on terminal Vaccinium branches of the ALMT family, we applied the codon-based branch-site test [17] implemented in CODEML, which is included in the PAML package v4.10 [29] to the three orthogroups that showed elevated within-Vaccinium Ka/Ks in the per-orthogroup analysis (Subfamily 5/OG0008930, fold-change 1.88×; Subfamily 4/OG0006223, 1.33×; Subfamily 2/OG0002701, 1.53×; all three reach Bonferroni significance at the per-OG Mann–Whitney U test, see Section 3.3).

For each focal orthogroup, the analysis pipeline was as follows. (1) Protein-guided codon alignment: amino-acid sequences translated from the longest ORF of each CDS were aligned with MAFFT v7.526 [20] using the L-INS-i algorithm (mafft-localpair-maxiterate 1000). The protein alignment was then back-translated to a codon alignment by mapping each aligned residue (or gap) to the corresponding three nucleotides of the source CDS, preserving the reading frame. Premature stop codons in any sequence triggered exclusion of that sequence; no such cases were observed in any of the three orthogroups. (2) Reference gene tree inference: a maximum-likelihood gene tree was reconstructed from each per-OG protein alignment with IQ-TREE 2.2.2.2 [22] under the JTT F + G4 substitution model with 1000 ultrafast bootstrap replicates (-bb 1000); the simpler +G4 rate model was used at the per-OG level because the within-OG sequence sets are small enough that more complex rate-heterogeneity models are not justified by BIC, in contrast to the family-wide JTT + F + R5 selection in Section 2.3. The resulting consensus tree was used as the fixed input topology for the PAML test. (3) Branch labelling: in the PAML input tree, all terminal branches leading to Vaccinium ALMTs were labelled as foreground (#1), and all remaining branches were left unlabelled as background. This foreground scheme tests whether lineage-specific positive selection occurred on individual Vaccinium ALMT paralogs after their separation from the most-recent shared ancestor with the non-Vaccinium outgroups; the shallow internal node connecting all Vaccinium leaves to their single common ancestor was deliberately left unlabelled, because that branch predates much of the Vaccinium divergence and labelling it would no longer isolate lineage-specific change. (4) Model comparison: for each orthogroup, two CODEML runs were performed using the branch-site Model A (NSsites = 2, model = 2) framework—the alternative model with foreground ω_2_ free to exceed 1 (fix_omega = 0, initial ω = 1.5), and the null model with foreground ω_2_ fixed at 1 (fix_omega = 1, omega = 1). All other CODEML settings followed PAML defaults for codon analysis. The likelihood-ratio test statistic, 2ΔlnL = 2 × (lnL_Alt − lnL_Null), was assessed against a 50:50 mixture of χ^2^_0_ and χ^2^_1_ distributions [17]; raw mixture-distribution *p*-values were further Bonferroni-corrected for the three OG-level tests performed (multiplier × 3). Sites under positive selection on the foreground lineage were identified using Bayes Empirical Bayes (BEB) inference [30], with a posterior probability (p_class2a + p_class2b) > 0.95 classifying a site as high confidence under positive selection. For Subfamily 2, the optimized log-likelihood of the alternative model was indistinguishable from that of the null at the precision of the CODEML convergence threshold, yielding a 2ΔlnL value that was effectively zero (reported as −0.08 from the unconstrained optimization; small negative values in nested models reflect numerical convergence noise and are treated here as evidence of no foreground signal rather than as a biologically meaningful negative LRT). The qualitative conclusion (no branch-site signal in Subfamily 2) was robust to multiple initial values of ω_2_ ∈ {0.5, 1.5, 5.0}, which all converged to indistinguishable lnL.

For the Subfamily 5 (OG0008930) analysis, the BEB-significant sites were further mapped onto the three-dimensional structure of the *Vaccinium duclouxii* GWHPDOBL013499 (Vdu1) reference protein predicted using AlphaFold2-ptm as implemented in ColabFold v1.5 (https://github.com/sokrypton/ColabFold; accessed on 20 April 2026) (see Section 2.7), permitting per-residue pLDDT readouts and Cα–Cα distance estimates. For Subfamily 4 (OG0006223), BEB-significant sites were assigned to topological regions of the Subfamily 4 *Vaccinium duclouxii* reference protein (GenBank GWHPDOBL014285) using DeepTMHMM v1.0 (https://dtu.biolib.com/DeepTMHMM; accessed on 20 April 2026) for transmembrane topology prediction; no AlphaFold2-ptm/ColabFold structural model was generated for the SF4 reference protein in this study.

### 2.7. Protein Structure Prediction and Topology Analysis

The three-dimensional structure of the Vaccinium duclouxii Subfamily 5 ALMT representative (Vdu1, 420 aa) was predicted using ColabFold v1.5 [31], which implements AlphaFold2-ptm with MMseqs2-based multiple sequence alignment construction. The unrelaxed model 1 (seed 000) was retained as the primary output. Per-residue confidence (pLDDT) was extracted from the B-factor column of the predicted PDB file, with values ≥ 90 classified as very high confidence, 70–<90 as confident, 50–<70 as low, and <50 as very low. Transmembrane helices were predicted independently using DeepTMHMM v1.0 [32] for all 145 retained ALMT loci, providing the primary topology assignment. For Vdu1, DeepTMHMM identified six transmembrane helices (residues 51–71, 75–95, 101–118, 127–147, 157–173, and 186–203), with the C-terminal cytoplasmic domain (CHD) spanning residues 204–420; this canonical six-transmembrane architecture is consistent with the experimentally determined cryo-EM structures of Arabidopsis ALMT1 [7] and Glycine max ALMT12 [8]. As a complementary screen, the Kyte–Doolittle hydropathy profile [33] was computed in parallel (sliding window 19 residues, threshold > 0.5, minimum segment length 17 aa). Across the family-wide 145-locus set, DeepTMHMM recovered the canonical six-TM ALMT architecture in 137 of 145 retained loci (94.5%), with the remaining 8 loci predicted at 4, 7, or 8 helices and treated as topology variants requiring experimental validation. The spatial location of BEB-significant sites was then determined by mapping each residue onto these segments using the codon alignment. Three-dimensional distances between BEB-positive residues were computed from Cα coordinates in the AlphaFold2-predicted model.

### 2.8. Synteny and Gene Duplication Mode Classification

To investigate the duplication history of *Vaccinium* and *Malus* ALMTs, intra- and inter-species synteny was inferred with MCScanX (https://github.com/wyp1125/MCScanX; accessed on 20 April 2026) [34]. All-versus-all protein BLAST (BLAST+ blastp, E < 1 × 10−10, max_target_seqs = 5) was performed within and among the three chromosome-level genomes in the dataset—M. domestica (GDT2T_hap1 [5], 17 chromosomes), Vaccinium duclouxii (T2T, 12 chromosomes), and Vaccinium darrowii (12 pseudochromosomes)—all of which were used for the duplication-mode classification and synteny analysis. Simplified GFF files (chromosome ID, gene ID, start, end) were generated for each species and merged for cross-species analysis. MCScanX was run with default parameters (MATCH_SCORE = 50, GAP_PENALTY = −1, MATCH_SIZE = 5, MAX_GAPS = 25). Each ALMT gene was classified into one of five duplication modes—singleton, dispersed, proximal, tandem, or whole-genome/segmental duplicate—using the bundled duplicate_gene_classifier tool.

### 2.9. Statistical Analysis

All statistical comparisons were performed in Python 3.11 with NumPy 1.26, pandas 2.2, and SciPy 1.13. Per-OG Ka/Ks distributions between groups were summarized as median, interquartile range, and number of pairs. Comparison between Vac–Vac and Out–Out Ka/Ks distributions was performed using the one-tailed Mann–Whitney U test (alternative = “greater”; method = “asymptotic” as implemented in scipy.stats.mannwhitneyu) on the raw pairwise Ka/Ks values from Supplementary Table S5. Raw *p*-values were Bonferroni-corrected for the six OG-level tests (multiplier × 6) and capped at 1.0; significance is reported as adjusted *p* < 0.05/<0.01/<0.001 in figure annotations. Fold-change between group medians is reported descriptively in addition to the test statistic. For the per-OG PAML branch-site tests, the likelihood-ratio test statistic 2ΔlnL = 2 × (lnL_Alt − lnL_Null) was assessed against a 50:50 mixture of χ^2^_0_ and χ^2^_1_ distributions [17], with raw mixture-distribution *p*-values further Bonferroni-corrected for the three OG-level tests performed (multiplier × 3).

## 3. Results

### 3.1. Genome-Wide Identification of ALMT Genes in 11 Angiosperm Species

Using a dual identification pipeline combining HMMSEARCH against the ALMT-family Pfam profile (PF11744; HMMER v3.3.2) and BLASTP queries with 55 validated AtALMT and MdALMT sequences (E < 1 × 10^−10^, identity > 30%, query coverage > 50%), followed by per-species CD-HIT redundancy reduction at 95% identity, length filtering (≥200 aa), HMMSEARCH re-validation against PF11744 with bit-score ≥ 50 and envelope coverage ≥ 60%, and locus-level deduplication of alternative transcripts to a single representative per Parent gene (Methods 2.2), we identified 145 non-redundant ALMT loci across the 11 angiosperm genomes (Figure 1; Supplementary Table S2). Family size varies markedly by species and phylogenetic context. The four diploid Vaccinium species harbour compact ALMT families of 8–10 members per genome (*Vaccinium darrowii*, eight; *Vaccinium macrocarpon*, 10; *Vaccinium microcarpum*, nine; *Vaccinium duclouxii*, nine; *Vaccinium corymbosum* tetraploid, 13). Their sister Ericaceae outgroup R. simsii contains 11 ALMTs. The two acid-tolerant outgroups carry larger families as follows: *A. eriantha* (Actinidiaceae) with 21 and C. sinensis (Theaceae) with 15 ALMTs, the latter being slightly fewer than the 16 members reported by Li et al. [11] under their less stringent HMM filter. The Rosaceae reference M. domestica harbours 24 locus-level ALMTs, broadly matching the 25 transcript-level entries reported by Ma et al. [4]; locus-level deduplication of alternative-transcript redundancy in our pipeline yields these 24 unique loci, all consistent with the T2T GDT2T_hap1 assembly used here [5]. *V. vinifera* (Vitaceae) contains 12 ALMTs, and *A. thaliana* contains 13 ALMT-family loci passing our unified HMM filter (Table S2). We use TAIR locus IDs (e.g., At1g08430, At5g46610) throughout the manuscript for the Arabidopsis entries because the in-house AtALMT numbering used in our pipeline does not strictly follow the canonical AtALMT1–14 nomenclature; the exact TAIR mapping is provided in Table S2. Per-species counts and edaphic preferences are illustrated in Figure 1; family size thus correlates with phylogenetic family and recent WGD history rather than with edaphic preference.

All 145 retained ALMT loci contain the PF11744 ALMT domain (bit-score ≥ 50, HMM coverage ≥ 60%; Supplementary Table S2). DeepTMHMM independently recovers the canonical six-transmembrane architecture in 137 of 145 loci (94.5%); the remaining eight loci with four, seven, or eight predicted helices are interpreted as topology variants requiring experimental validation. The family spans a three-fold range in size across the 11 species. The largest families are concentrated in lineages with extensive WGD history: *M. domestica* (24 locus-level ALMTs) reflects the apple-specific whole-genome duplication documented by Velasco et al. [6], while *A. eriantha* (21) and *C. sinensis* (15) carry intermediate-sized families consistent with their own family-specific WGD histories. The four smallest families are concentrated within the diploid Vaccinium species themselves (*Vaccinium darrowii* eight, *Vaccinium duclouxii* nine, *Vaccinium microcarpum* nine, and *Vaccinium macrocarpon* 10), with the tetraploid *Vaccinium corymbosum* (13) intermediate. Critically, the four acid-tolerant lineages sampled here (*Vaccinium*, *Rhododendron*, *Camellia*, and *Actinidia*) span almost the entire family-size range, ruling out any simple correlation between edaphic preference and ALMT gene number.

### 3.2. Phylogenetic Structure and Orthogroup Assignment

**Figure 2:**
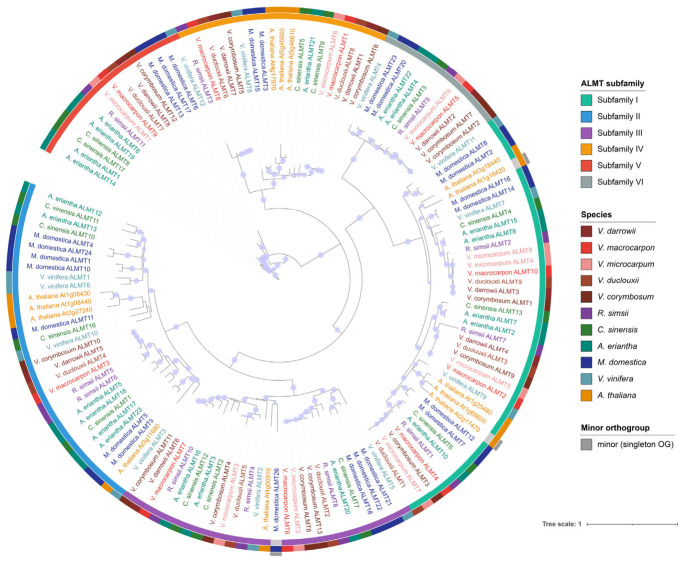
Maximum-likelihood phylogeny of 145 ALMT loci from 11 angiosperm species, midpoint-rooted. Filled circles indicate ultrafast-bootstrap support ≥ 70 (size proportional to value), while light gray circles indicate ultrafast-bootstrap support < 70. The inner ring labels the OrthoFinder-defined subfamily (I–VI); the outer ring labels the species. Leaves follow “Genus species ALMTn” nomenclature.

Maximum-likelihood phylogenetic inference on the trimmed 326-column alignment of all 145 locus-level ALMTs recovered six major clades with strong ultrafast-bootstrap support (Figure 2), in close concordance with OrthoFinder-defined orthogroups (Supplementary Table S2). The six clades correspond to OG0001431 (38 members), OG0002701 (31), OG0001688 (26), OG0006223 (19), OG0008930 (16), and OG0012384 (12), collectively containing 142 (98%) of all 145 ALMT loci. We hereafter refer to these as Subfamilies 1–6 (in decreasing size). All five Vaccinium species are represented in all six subfamilies, indicating that the six major ALMT subfamilies were present in the common ancestor of Vaccinium and have been retained throughout the genus. The absence of Vaccinium-specific orthogroups argues against gene-family expansion as the mechanism of acid-soil adaptation in this lineage.

Per-subfamily species distribution further refines this picture. Each diploid Vaccinium species contributes one to four paralogs per major subfamily (Supplementary Table S2), reflecting either single-copy retention or limited tandem/dispersed duplication of each ancestral subfamily. Beyond the six major subfamilies, three of the 145 ALMT loci were assigned to minor orthogroups (each represented by only a single ALMT in this dataset) and were excluded from per-orthogroup comparative analyses but retained in the master sequence list. These minor groups most likely represent recent species-specific paralogs or annotation-borderline ALMT-like proteins that have diverged sufficiently from the canonical subfamilies to escape OrthoFinder clustering. Branch support across the 145-leaf tree is consistently higher within subfamilies than at the deep nodes that separate them, a pattern compatible with rapid early diversification of the ancestral angiosperm ALMT family followed by extensive lineage-specific copy-number evolution within each subfamily.

### 3.3. Subfamily-Specific Elevation of Ka/Ks in Within-Vaccinium Pairwise Comparisons

**Figure 3:**
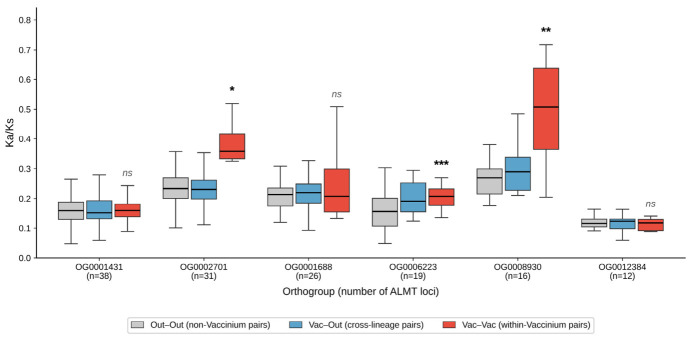
Subfamily-specific *Ka/Ks* elevation in within-Vaccinium pairwise comparisons. Pairwise Ka/Ks for all within-orthogroup gene pairs across the six major ALMT subfamilies (n = total loci per orthogroup), partitioned into Out–Out (grey), Vac–Out (blue), and Vac–Vac (red) comparisons. Significance symbols above each Vac–Vac box: one-tailed Mann–Whitney U test (scipy.stats.mannwhitneyu, method = “asymptotic”) versus Out–Out, Bonferroni-corrected over the six OG-level tests (multiplier × 6). Three orthogroups show Bonferroni-significant within-Vaccinium elevation: OG0006223 (1.33×, adjusted *p* = 5.16 × 10^−4^; ***), OG0008930 (1.88×, adjusted *p* = 1.49 × 10^−3^; **), and OG0002701 (1.53×, adjusted *p* = 1.04 × 10^−2^; *). Significance code: *** *p* < 0.001; ** *p* < 0.01; * *p* < 0.05; *ns*, not significant.

Pairwise Ka/Ks values were computed for all gene pairs within each of the six subfamilies and partitioned into Vac–Vac, Out–Out, and Vac–Out comparisons (Figure 3; Supplementary Table S5). Vac–Vac Ka/Ks distributions are elevated relative to Out–Out in three of the six subfamilies and remain significant after Bonferroni correction across the six *OG-level tests* as follows: Subfamily 5 (OG0008930, fold-change 1.88× over non-Vac background; raw *p* = 2.49 × 10^−4^; Bonferroni-adjusted *p* = 1.49 × 10^−3^), Subfamily 4 (OG0006223, 1.33×; raw *p* = 8.60 × 10^−5^; adjusted *p* = 5.16 × 10^−4^), and Subfamily 2 (OG0002701, 1.53×; raw *p* = 1.73 × 10^−3^; adjusted *p* = 1.04 × 10^−2^). The remaining three subfamilies show no significant within-Vaccinium elevation after correction (OG0001431/SF1, 1.00×, adjusted *p* = 0.653; OG0001688/SF3, 0.97×, adjusted *p* = 1.000; OG0012384/SF6, 1.02×, adjusted *p* = 1.000). Importantly, all 1731 within-OG pairwise Ka/Ks values fall below 1 (range 0–0.925), consistent with overall purifying selection at the family level; the lineage-specific positive selection detected at individual codon sites by the branch-site tests (Section 3.4) is reported here as a complementary site-level signal that does not contradict this family-wide purifying-selection profile. Subfamilies 5 and 4 thus emerge as the orthogroups with the strongest signals of lineage-associated divergence in within-Vaccinium pairwise comparisons and were taken forward to codon-based branch-site testing alongside Subfamily 2.

Inspection of the underlying pairwise distributions (Supplementary Table S5) indicates that the within-Vaccinium Ka/Ks elevation in Subfamilies 5, 4, and 2 is not driven by a single outlier pair: each elevated subfamily contains multiple within-Vaccinium comparisons that span both within-section (V. corymbosum/Vaccinium darrowii, section Cyanococcus) and between-section (Vaccinium macrocarpon/Vaccinium microcarpum, section Oxycoccus) pairings. Across all six subfamilies the median Out–Out Ka/Ks is consistently below 0.3, indicating that most ALMT pairs in our dataset are under strong purifying selection; the elevated Vac–Vac signal in Subfamilies 5, 4, and 2 is therefore a localized relaxation of constraint, with focal positive-selection signals subsequently detected for Subfamilies 5 and 4 in the branch-site tests (Section 3.4) and Subfamily 2 reframed as relaxed selective constraint without per-site positive selection. The number of Vac–Vac pairs available per OG is necessarily limited by the size of the Vaccinium contribution to each orthogroup (*Vac–Vac n* = 105 for SF1, 55 for SF3, 35 for SF4, 10 for SF5, 10 for SF6, and six for SF2), which constrains the statistical power of the asymptotic Mann–Whitney U test for the smaller orthogroups; the corresponding raw and Bonferroni-adjusted *p*-values reported above and in Table 2 should therefore be interpreted in conjunction with the per-OG pair counts and fold-change estimates.

### 3.4. PAML Branch-Site Tests Detect Candidate Positively Selected Sites on Terminal Vaccinium Branches in Subfamilies 5 and 4

PAML branch-site tests (Model A vs. null A) [17] were performed on the three subfamilies with within-Vaccinium Ka/Ks elevation that reached Bonferroni-significance at the per-OG Mann–Whitney U test (Section 3.3) as follows: Subfamily 5 (OG0008930, 16 loci), Subfamily 4 (OG0006223, 19 locus-deduplicated loci), and Subfamily 2 (OG0002701, 31 loci). All terminal Vaccinium branches in each per-OG gene tree (IQ-TREE 2.2.2.2 consensus tree, JTT + F + G4) were labelled as foreground (#1); all other branches were left unlabelled as background. The likelihood-ratio test statistic 2ΔlnL = 2 × (lnL_Alt − lnL_Null) was assessed against a 50:50 mixture of χ^2^_0_ and χ^2^_1_ distributions, with raw mixture-distribution *p*-values Bonferroni-corrected across the three OG-level tests (multiplier × 3). Sites under positive selection on the foreground lineage were identified by Bayes Empirical Bayes (BEB) inference [30] with a posterior threshold of p(class 2a) + p(class 2b) > 0.95.

**Figure 4:**
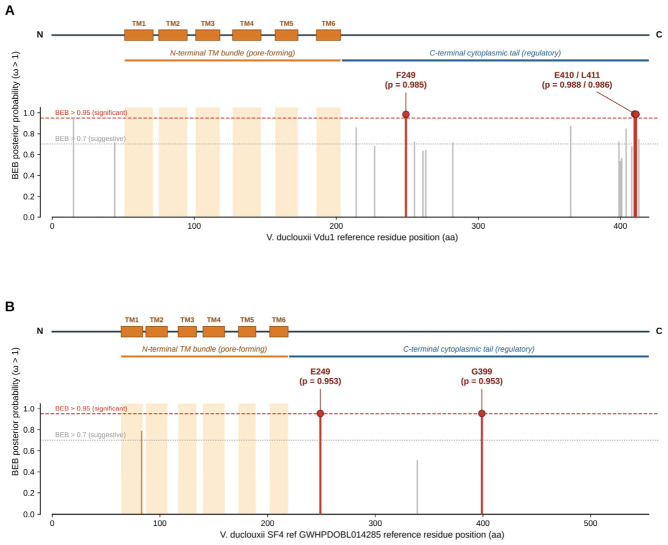
PAML branch-site tests detect candidate positively selected sites on terminal Vaccinium branches in Subfamilies 5 and 4. (**A**) Subfamily 5 (OG0008930; 16 ALMT loci): likelihood-ratio test 2ΔlnL = 40.65, raw mixture-distribution p = 9.1 × 10^−11^, Bonferroni-adjusted p = 2.7 × 10^−10^. The following three codons exceed BEB > 0.95 (red filled circles): F249 (0.985), E410 (0.988), and L411 (0.986), referenced to the Vaccinium duclouxii Vdu1 protein (420 aa; Supplementary Table S6). Vdu1 DeepTMHMM topology places TM1–TM6 in residues 51–203 and the C-terminal cytoplasmic domain in residues 204–420; the three SF5 sites all lie in the cytoplasmic domain downstream of TM6. (**B**) Subfamily 4 (OG0006223; 19 locus-deduplicated ALMT loci): 2ΔlnL = 12.95, raw mixture-distribution *p* ≈ 1.6 × 10^−4^, Bonferroni-adjusted *p* ≈ 4.8 × 10^−4^. Two codons exceed BEB > 0.95: E249 (0.953) and G399 (0.953), referenced to the *Vaccinium duclouxii* SF4 representative protein (GenBank GWHPDOBL014285; 554 aa). Both SF4 sites map to the C-terminal cytoplasmic domain of the SF4 reference protein under DeepTMHMM topology. Pale yellow vertical bands indicate predicted transmembrane regions, and dashed horizontal lines indicate BEB posterior-probability thresholds.

**Figure 5:**
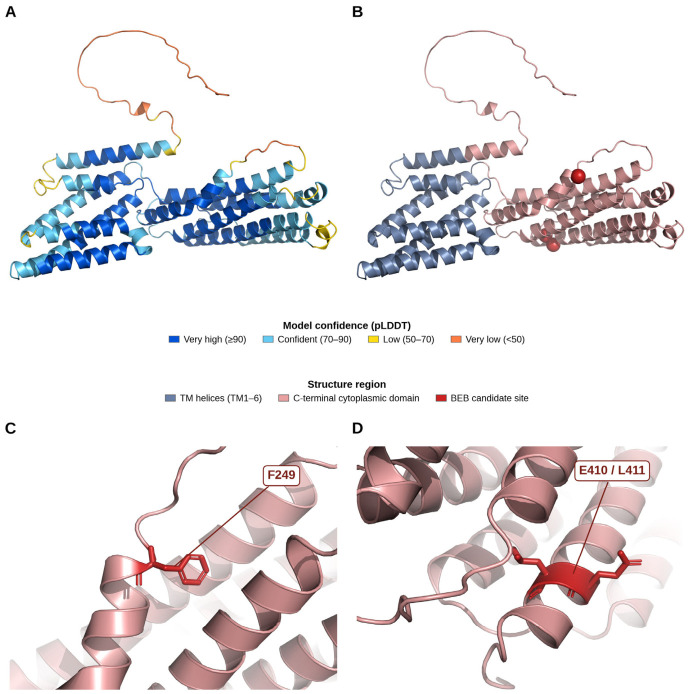
AlphaFold2-predicted structure of Vdu1 (Subfamily 5 representative, 420 aa) with mapped BEB-significant sites. (**A**) Overall structure coloured by per-residue pLDDT (blue, very high ≥ 90; cyan, confident 70–<90; yellow, low 50–<70; orange, very low < 50); BEB-significant residues shown as red spheres. (**B**) Same view coloured by DeepTMHMM topology: TM helices TM1–TM6 (slate, residues 51–203), C-terminal cytoplasmic domain (salmon, residues 204–420); F249 and E410/L411 highlighted as red spheres. (**C**) Close-up of F249 in the upstream cytoplasmic-domain region (residues ~245–253 shown as sticks), above the KEVDEL motif. (**D**) Close-up of E410 and L411 within the conserved C-terminal KEVDEL motif (residues ~406–414 shown as sticks); E410 and L411 form an adjacent pair 3.8 Å apart (Cα–Cα).

For Subfamily 5 (16 ALMT loci, OG0008930), the test detected highly significant positive selection on terminal Vaccinium branches (Figure 4A): 2ΔlnL = 40.65 (df = 1; raw mixture-distribution *p* = 9.1 × 10^−11^; Bonferroni-adjusted *p* = 2.7 × 10^−10^), with the alternative model recovering a foreground ω_2_ ≈ 34.95 in 5.6% of sites. The following three codons exceeded the BEB > 0.95 posterior-probability threshold: F249 (BEB = 0.985), E410 (0.988), and L411 (0.986), where positions refer to the Vaccinium duclouxii Vdu1 reference (420 aa). AlphaFold2 per-residue pLDDT at these positions was 78.1 (F249), 92.6 (E410), and 93.4 (L411), reflecting confident local prediction at the cytoplasmic loop and very-high confidence at the C-terminal pair (Supplementary Table S6). All three SF5 BEB-significant sites lie within the C-terminal cytoplasmic domain (CHD) of Vdu1 (residues 204–420) downstream of TM6 as resolved by DeepTMHMM [32]; E410 and L411 form an adjacent pair within a conserved C-terminal acidic motif (“KEVDEL”), while F249 lies in an upstream cytoplasmic-domain stretch above this motif (Figure 5).

For Subfamily 4 (19 locus-deduplicated ALMT loci, OG0006223), the test was also significant on terminal Vaccinium branches (Figure 4B): 2ΔlnL = 12.95 (df = 1; raw mixture-distribution *p* ≈ 1.6 × 10^−4^; Bonferroni-adjusted *p* ≈ 4.8 × 10^−4^). Bayes Empirical Bayes identified the following two sites exceeding the 0.95 posterior threshold: E249 (BEB = 0.953) and G399 (BEB = 0.953), referenced to the *Vaccinium duclouxii* Subfamily 4 representative protein (GenBank GWHPDOBL014285; 554 aa). The two SF4 BEB-significant sites both map to the C-terminal cytoplasmic domain of the SF4 reference protein under DeepTMHMM topology. We emphasize that this SF4 mapping is based on DeepTMHMM topology assignment alone: unlike the SF5 sites, the SF4 sites were not mapped onto an AlphaFold2 three-dimensional model and no per-residue pLDDT or Cα–Cα distance estimates are reported here.

For Subfamily 2 (31 loci, OG0002701), the test yielded no evidence for terminal-Vaccinium positive selection: 2ΔlnL = −0.08 (raw mixture-distribution p = 0.5; Bonferroni-adjusted p = 1.0); no BEB site exceeded the 0.95 threshold. The elevated within-Vaccinium pairwise Ka/Ks observed for Subfamily 2 in Section 3.3 is therefore interpreted as relaxed selective constraint on the Vaccinium SF2 paralogs rather than as evidence of positive selection on a specific Vaccinium foreground branch. Together, the branch-site analyses distinguish a focal pattern in Subfamilies 5 and 4 (Bonferroni-significant LRTs and individually localisable BEB residues mapped to cytoplasmic regulatory regions, with three-dimensional structural mapping available only for SF5) from a non-significant pattern in Subfamily 2.

The contrast between Subfamilies 5 and 4 (Bonferroni-significant LRTs with individually localisable BEB-supported residues) and Subfamily 2 (no branch-site signal in the locus-deduplicated data, with elevated pairwise Ka/Ks interpreted as relaxed selective constraint) suggests two distinct evolutionary regimes within the Vaccinium ALMT family as follows: focal episodes of candidate positive selection at five BEB-supported residues distributed across two cytoplasmic regulatory regions (three SF5 sites on Vdu1; two SF4 sites on the SF4 reference protein), and an absence of branch-site signal in Subfamily 2 whose elevated within-Vaccinium pairwise Ka/Ks is therefore attributed to relaxed selective constraint rather than positive selection. For Subfamily 5 specifically, the alternative-model background ω in site classes 0 and 1 remained constrained to 0.19 and 1.00 respectively, confirming that the elevated foreground ω_2_ ≈ 34.95 is restricted to the Vaccinium foreground branches and does not reflect a genome-wide change in selective regime; this SF5-specific ω_2_ value should not be extrapolated to Subfamily 4 (foreground site-class proportions and ω_2_ for SF4 are reported in Supplementary Data S4). Inspection of the codon alignment further indicates that all three SF5 BEB-significant sites (F249, E410, L411) are conserved within Vaccinium but differ from the non-Vaccinium consensus at each position, consistent with a Vaccinium-associated residue pattern at these codon positions; the two SF4 BEB-significant sites (E249, G399) show the same alignment-level pattern in the SF4 codon alignment (Supplementary Data S3b).

### 3.5. Vaccinium Retains the Majority of Ancestral ALMT Loci but Few Intra-Species Syntenic Paralogs

**Figure 6:**
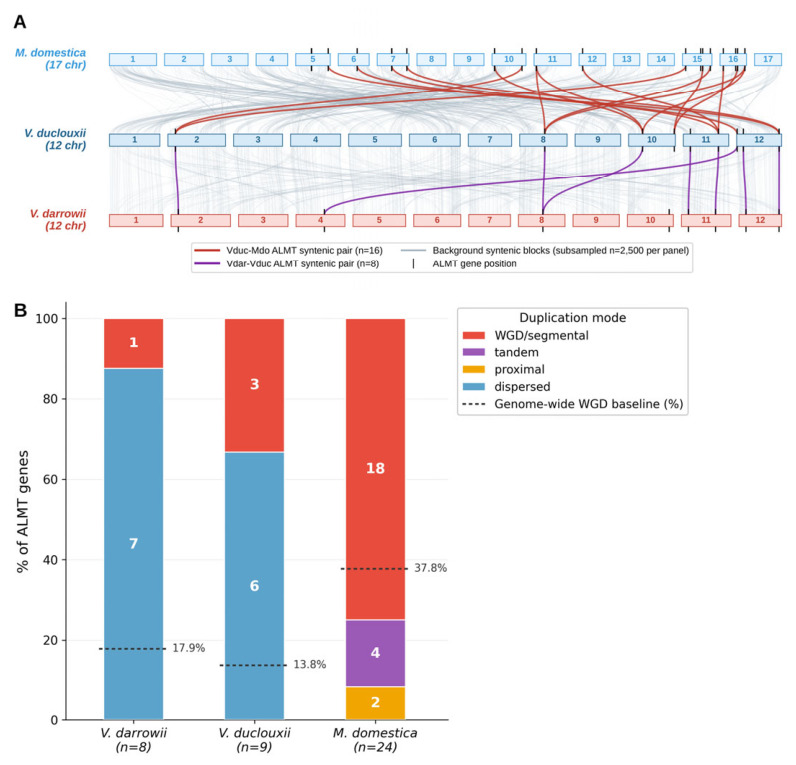
Lineage-specific duplication modes and syntenic conservation of ALMT genes. (**A**) Cross-species synteny across *M. domestica* (top), *Vaccinium duclouxii* (middle), and *Vaccinium darrowii* (bottom). Red lines: Vdu–Md ALMT syntenic pairs (*n* = 16); purple lines: Vda–Vdu pairs (*n* = 8); grey: background syntenic blocks (*n* = 2500 subsampled per panel). (**B**) Duplication-mode classification of all ALMTs by MCScanX. Dotted lines indicate the genome-wide percentage of WGD/segmental duplicates per species (*Vaccinium darrowii* 17.9%, *Vaccinium duclouxii* 13.8%, *M. domestica* 37.8%).

MCScanX synteny analysis of the three chromosome-level genomes in our dataset (*M. domestica*, *Vaccinium duclouxii*, and *Vaccinium darrowii*) revealed two complementary patterns of ALMT distribution (Figure 6). At the cross-species level, seven of the nine VduALMT loci participated in 16 Vdu–Md syntenic pairings, indicating extensive retention of the ancestral ALMT gene order between Vaccinium and *M. domestica*. At the intra-species level, however, the two species differ markedly. *M. domestica* contains multiple intra-species ALMT syntenic paralog pairs reflecting the Rosaceae-specific whole-genome duplication, whereas *Vaccinium duclouxii* contains one intra-species ALMT syntenic pair and *Vaccinium darrowii* contains 0 (Supplementary Table S7). Per-species duplication-mode classification (Figure 6B) recapitulates this contrast: the majority of MdALMTs are WGD/segmental duplicates (18 of 24 = 75%), whereas 88% of *Vaccinium darrowii* ALMTs (7/8) and 67% of *Vaccinium duclouxii* ALMTs (6/9) are dispersed (Supplementary Table S7). Vaccinium ALMTs thus reflect compact retention of the ancestral syntenic complement with limited intra-species post-WGD duplication, in marked contrast to the apple ALMT family. As a conservative caveat: the *V. corymbosum*, *Vaccinium macrocarpon*, *Vaccinium microcarpum*, and R. simsii assemblies are not yet chromosome-level and were not included in the MCScanX analysis; the synteny conclusions above therefore apply specifically to the three chromosome-level Md/Vdu/Vda comparison and should not be extrapolated unconditionally to the broader Vaccinium genus.

Within the genus, the chromosome-level genomes of *Vaccinium duclouxii* and *Vaccinium darrowii* share extensive ALMT synteny: 8 of 9 VduALMTs lie in syntenic blocks with *Vaccinium darrowii* orthologs (Vda–Vdu syntenic pairs *n* = 8; Figure 6A), reflecting the close phylogenetic relationship between the two species and extensive retention of ancestral synteny in the absence of recent intra-genus WGD. The single intra-species syntenic ALMT paralog pair in *Vaccinium duclouxii* and the absence of such pairs in *Vaccinium darrowii* indicate very limited retained intra-species ALMT collinearity in the two chromosome-level Vaccinium genomes (Supplementary Table S7); this observation reflects the present-day distribution of syntenic ALMT pairs and is not interpreted here as a precise statement about the timing of duplication or loss. The duplication-mode contrast is also reflected in the per-species genome-wide WGD baseline: *M. domestica*’s 37.8% WGD-derived genome-wide background is reduced to 13.8% in *Vaccinium duclouxii* and 17.9% in *Vaccinium darrowii* (Figure 6B), and the ALMT family in both Vaccinium species sits closer to their own lower genome-wide baselines than to the apple baseline (*Vaccinium darrowii* ALMT WGD/segmental 12.5% vs. 17.9% baseline; *Vaccinium duclouxii* ALMT WGD/segmental 33.3% vs. 13.8% baseline—i.e., the *Vaccinium duclouxii* ALMT family is enriched ~2.4× over its own genome-wide WGD background, but its absolute fraction remains well below the apple ALMT family).

## 4. Discussion

### 4.1. Vaccinium Follows a Compact-Retention Strategy Rather than Family Expansion

Our comparative genomic analysis demonstrates that the ALMT family in *Vaccinium* follows a strikingly compact-retention strategy. Diploid Vaccinium species harbour only 8–10 ALMTs each—fewer than half the size of the *M. domestica* family (24 locus-level ALMTs) and substantially fewer than the acid-tolerant outgroup *A. eriantha* (21). This compact family size, combined with high cross-species syntenic retention and the near-complete absence of intra-species syntenic paralogs (0–1 pairs in *Vaccinium duclouxii*/*Vaccinium darrowii* versus multiple WGD-derived pairs in *M. domestica*), points to a pattern of lineage-specific paralog loss rather than failure to acquire paralogs in the first place. The pattern is best interpreted as post-WGD diploidisation: *Vaccinium* and *M. domestica* share the eudicot γ hexaploidisation event, but only the Rosaceae lineage retained the more recent σ/δ-class WGD [6], and *Vaccinium* subsequently lost most of the duplicated ALMT paralogs that an ancestral hexaploid would have provided. The compact configuration is consistent with hypothesis H2 (compact retention with divergence) and inconsistent with hypothesis H1 (expansion-driven adaptation).

This pattern of compact retention is consistent with the broader genome-scale duplicate-mode profile of the *Vaccinium* genus. Genome-wide duplicate classifications of the *Vaccinium duclouxii* reference [16] show a substantially lower fraction of WGD-derived genes than *M. domestica* or other recently duplicated Rosaceae taxa, indicating that the absence of intra-species *ALMT* paralogs reported here is not an *ALMT*-specific anomaly but reflects a genus-level diploidisation pattern. Under the dosage-balance framework, loss of paralogs after WGD is expected to be biassed away from genes encoding subunits of obligate large multimeric complexes (transcription factors, ribosomal proteins, and chromatin machinery) and toward genes whose products function autonomously or as small homo-oligomers. *ALMT* channels assemble as homodimers at the plasma membrane and act as individual transporters rather than as members of large protein complexes [7,8], and the family is therefore expected to be relatively tolerant of paralog loss. The pattern we observe—every *Vaccinium* species retains representatives of all six major *ALMT* subfamilies, but with one to four paralogs per subfamily rather than the multiple WGD-derived paralogs seen in apple—is precisely the signature expected of dosage-balance-permitted paralog loss against a backdrop of subfamily-level functional conservation.

### 4.2. Lineage-Specific Positive Selection Is Concentrated on Cytoplasmic Regulatory Regions Rather than the Pore-Lining Transmembrane Helices

Branch-site tests detected significant positive selection on terminal *Vaccinium* branches in two of the three tested subfamilies (Subfamily 5/OG0008930: 2ΔlnL = 40.65, Bonferroni-adjusted *p* = 2.7 × 10^−10^, three BEB-significant sites; Subfamily 4/OG0006223: 2ΔlnL = 12.95, Bonferroni-adjusted *p* ≈ 4.8 × 10^−4^, two BEB-significant sites), with a non-significant result for Subfamily 2 (2ΔlnL = −0.08, Bonferroni-adjusted *p* = 1.0). The five BEB-significant residues across Subfamilies 5 and 4—F249, E410, L411 (SF5, on *Vaccinium duclouxii* Vdu1), E249, G399 (SF4, on the *Vaccinium duclouxii* SF4 representative GWHPDOBL014285)—map to the C-terminal cytoplasmic regulatory regions of their respective reference proteins rather than to the transmembrane pore-lining helices that determine ion conductance (Figure 4 and Figure 5). Below, we discuss the SF5 and SF4 candidates in turn, with attention to the different lines of structural evidence available for the two subfamilies.

For Subfamily 5, the three BEB-significant sites were mapped onto the AlphaFold2 model of *Vaccinium duclouxii* Vdu1 (Figure 5; per-residue pLDDT scores reported in Supplementary Table S6). F249 sits in the upstream portion of the C-terminal cytoplasmic domain (CHD) above the conserved C-terminal acidic motif; this CHD region has been implicated in voltage gating and Ca^2+^/calmodulin binding in the related QUAC1/ALMT12 channel of Arabidopsis [9] and Brachypodium distachyon ALMT12 [10]. E410 and L411 form an adjacent pair within a conserved C-terminal acidic motif (“KEVDEL”) in the cytoplasmic domain, a broader region implicated in ligand-dependent channel regulation by the available ALMT structures of Arabidopsis ALMT1 [7] and Glycine max ALMT12 [8]; we do not claim that the KEVDEL motif itself has been directly demonstrated as a ligand-dependent gating locus, but locate it within the cytoplasmic regulatory module identified by those structures. The Cα–Cα distance between E410 and L411 in the AlphaFold2 model is 3.8 Å, and per-residue pLDDT exceeds 90 in this segment, supporting confident local structural prediction; in contrast, F249 lies in a less well-modelled cytoplasmic loop (pLDDT 78.1) and its local geometry should be treated as a confident-but-not-very-high-confidence prediction.

For Subfamily 4, the structural evidence is currently limited to topology assignment: under the DeepTMHMM topology of the *Vaccinium duclouxii* SF4 representative protein (GenBank GWHPDOBL014285; 554 aa), both BEB-significant sites E249 and G399 lie downstream of TM6 in the C-terminal cytoplasmic domain. No AlphaFold2 model of the SF4 reference protein was generated in this revision, and we therefore do not report Cα–Cα distances or per-residue pLDDT for the SF4 sites. We are deliberately conservative on this point: although the SF4 sites occupy a topologically equivalent compartment (C-terminal CHD downstream of TM6) to the SF5 sites, the absence of an SF4 three-dimensional model means that the SF4 candidates have not been examined at the same level of structural detail as the SF5 candidates. We do not claim that E249 and G399 lie within an experimentally characterized regulatory motif; this assignment is at the level of subdomain rather than residue-level geometry.

Taken together, the SF5 and SF4 findings concentrate the lineage-associated divergence in the *Vaccinium* ALMT family on the cytoplasmic side of the channel rather than on the transmembrane pore-forming bundle, consistent with the biochemistry of plant ALMT channels: the TM bundle determines anion permeability and selectivity and is typically constrained, whereas the CHD and its associated cytoplasmic loops mediate ligand-dependent gating, voltage sensing, and modulation by cytoplasmic partners and can be more readily tuned without disrupting core transport [7,8,9,10]. We emphasize the boundaries of this interpretation: the five BEB-supported residues are candidates emerging from a comparative-genomic analysis, not experimentally validated functional residues. Functional consequences must be tested by site-directed mutagenesis of each residue to its non-Vaccinium consensus, heterologous expression in oocytes or HEK293 cells, and direct electrophysiological characterization, complemented by screening of sequence variation at these positions across *Vaccinium* germplasm. The five candidate residues do not, by themselves, constitute a demonstrated molecular mechanism of acid-soil adaptation in Vaccinium.

### 4.3. Implications for Acid-Soil Adaptation and Limitations

The most economical interpretation of these findings is that *Vaccinium* acid-soil adaptation may have involved sequence divergence in regulatory regions of two ALMT subfamilies rather than expansion of the ALMT toolkit. This is biochemically plausible: malate efflux from *Vaccinium* roots into the acidic rhizosphere need not scale with the number of ALMT genes, but with the responsiveness of the existing ALMT channels to the pH, ionic, and metabolic signals that characterize an acidic rhizosphere environment. The five BEB-supported residues identified here (SF5: F249, E410, L411 on Vdu1; SF4: E249, G399 on the SF4 reference) define candidate positions whose functional consequences are directly testable. Among the five, the three SF5 sites are immediately tractable for structure-guided mutagenesis because each has been assigned a position on the AlphaFold2 model of Vdu1 with per-residue pLDDT readout; site-directed mutagenesis to the non-Vaccinium consensus (which differs at all three sites) followed by heterologous expression and electrophysiology in Xenopus oocytes or HEK293 cells would establish whether these substitutions alter channel gating, malate dependence, or pH sensitivity. The two SF4 sites are equally interesting evolutionary candidates but first require an independent structural model of the SF4 reference protein and complementary screening of sequence variation at these positions across Vaccinium germplasm to define the most informative functional experiment.

We note four limitations of the present analysis. First, the structural inferences in this study rely on AlphaFold2 predictions rather than experimentally determined coordinates, and they are available only for the SF5 reference protein (Vdu1); the SF4 site assignments rest on DeepTMHMM topology and have not been examined at three-dimensional resolution. Although the Vdu1 AlphaFold2 model has a mean pLDDT of 80.5 and the C-terminal region containing E410/L411 has pLDDT > 90, the assigned transmembrane topology and the precise geometry of the C-terminal motif should be regarded as predictions that require experimental validation. Second, the branch-site framework can be sensitive to alignment quality, foreground labelling, and the underlying gene-tree topology, and false-positive rates can be non-trivial under certain mis-specification conditions [17]. The strong signal in Subfamily 5 (2ΔlnL = 40.65) and the more moderate but Bonferroni-significant signal in Subfamily 4 (2ΔlnL = 12.95) are unlikely to be stochastic artefacts, but the formal demonstration that a given substitution is adaptive on the *Vaccinium* lineage will require functional characterization. Third, the obligate acidophilic phenotype of *Vaccinium* plants involves multiple physiological systems—iron acquisition, ammonium preference, nitrification suppression in the rhizosphere, and ericoid mycorrhizal associations—and the contribution of ALMT-mediated malate efflux to this phenotype, while plausible, has not been quantified at the whole-plant level. Fourth, the five *Vaccinium* species sampled here represent a small subset of the > 450 recognized species in the genus, and the Vaccinium-specific signal may not generalize uniformly across the genus. We further note that the foreground ω_2_ estimate for Subfamily 5 (≈34.95) lies at the upper end of values reported for plant ion-channel families, likely reflecting the small foreground partition; the strength of the likelihood-ratio signal (2ΔlnL = 40.65) nonetheless argues against a purely stochastic artefact, and leave-one-out and alternative-topology sensitivity analyses would further substantiate it.

These limitations indicate the most informative next steps. Site-directed mutagenesis of the three SF5 candidate residues (F249, E410, and L411) to their non-*Vaccinium* consensus, followed by heterologous expression and electrophysiological characterization in Xenopus oocytes or HEK293 cells, would directly test whether these residues alter channel gating, malate dependence, or pH response. For the two SF4 candidates (E249, G399), an independent AlphaFold2 model of the SF4 reference protein followed by the same mutagenesis-and-electrophysiology pipeline would extend the same functional analysis to the SF4 subfamily. Comparative expression profiling of the *Vaccinium* Subfamily 5 and Subfamily 4 members under acidic versus near-neutral rhizosphere conditions would clarify whether transcriptional responsiveness contributes to the acid-soil phenotype alongside the selection signals documented here. Expanding the sampling to include other Ericaceae genera (Rhododendron, Erica, and Calluna) and to additional *Vaccinium* species, particularly the non-blueberry section Oxycoccus, would test whether the Subfamily 5 and 4 signals are *Vaccinium*-specific or shared more broadly across acidophilic Ericaceae. Finally, integration of the ALMT data with rhizosphere microbiome and root-exudate profiles would help establish whether the malate-efflux capacity implied by the structural conservation is in fact deployed by *Vaccinium* roots in their characteristic acidic edaphic context.

Beyond *Vaccinium*, the analytical framework presented here—combining family-size variation, lineage-specific *Ka/Ks* elevation, codon-based branch-site testing, and structural mapping—provides a generally applicable template for identifying candidate residues underlying adaptive variation in plant ion-channel families. Crops and wild relatives such as tea (*Camellia sinensis*), buckwheat (*Fagopyrum* spp.), rye (*Secale cereale*), and the broader Ericaceae beyond *Vaccinium* all face comparable acid- or aluminum-rich edaphic conditions, and comparative-genomic profiling of their ALMT families using the same workflow would test whether the regulatory-tuning hypothesis suggested here for *Vaccinium* represents a recurrent solution to acid-soil adaptation across angiosperms or a *Vaccinium*-specific signal. From a translational perspective, the five sites identified across Subfamilies 5 (F249, E410, L411) and 4 (E249, G399) define a tractable starting point for marker-based screening in *Vaccinium* breeding populations; the three SF5 sites are immediately amenable to structure-guided functional validation, whereas the two SF4 sites first require independent structural modelling of the SF4 reference protein. Direct functional validation of even one of these five sites would convert a comparative-genomic candidate list into actionable selection targets.

For commercial *Vaccinium* production on acidic, aluminum-rich soils—which encompass much of the Pacific Northwest, southern China, Chilean Patagonia, and other major blueberry-growing regions—these findings offer a starting point for breeder-level molecular screening, with the explicit caveat that the candidate residues have not yet been functionally validated. Cultivar-level variation in aluminum tolerance has been documented metabolomically and physiologically in highbush blueberry [35], with resistant cultivars distinguished by enhanced organic-acid exudation and antioxidant capacity. The five candidate sites described here (SF5: F249, E410, L411; SF4: E249, G399) provide targets for screening sequence variation across *Vaccinium* germplasm and for testing association with cultivar-level aluminum tolerance, in conjunction with established phenotypic Al-tolerance assays based on root growth, organic-acid exudation, and photochemical performance. The three SF5 sites are priority targets for the first round of validation because of their AlphaFold2-mapped positions on the Vdu1 model; the two SF4 sites complement this set as broader evolutionary candidates whose functional significance will need to be tested following independent structural modelling of the SF4 reference protein. We do not claim that any of these sites is currently a validated marker for marker-assisted selection.

## 5. Conclusions

In this comparative genomic analysis of the ALMT gene family across 11 angiosperm species, we characterized the evolutionary trajectory of organic-acid efflux channels in *Vaccinium*, a genus of obligate acidophilic plants whose acid-soil adaptation has not previously been examined at the molecular-evolutionary level. The 145 non-redundant locus-level ALMT loci identified across the 11 species partition into six major subfamilies. All six subfamilies are represented in *Vaccinium*, yet each *Vaccinium* species harbours only one to four paralogs per subfamily. This results in a highly compact configuration of 8–10 total members per genome, which stands in marked contrast to the 24 locus-level ALMTs identified in *M. domestica*. Synteny analysis further established that *Vaccinium* retains the majority of ancestral ALMT loci shared with apple (seven of nine VduALMT loci participate in 16 Vdu–Md syntenic pairings) while showing very limited intra-species syntenic ALMT paralogs (*Vaccinium duclouxii*: 1 pair; *Vaccinium darrowii*: 0), based on the two chromosome-level *Vaccinium* genomes (*Vaccinium duclouxii* and *Vaccinium darrowii*) currently available.

In keeping with this compact configuration, three of the six subfamilies exhibit elevated nonsynonymous substitution rates in within-Vaccinium pairwise comparisons. Codon-based branch-site testing of these three orthogroups recovered Bonferroni-significant likelihood-ratio signals in Subfamilies 5 and 4, each with individually localisable BEB-supported candidate sites as follows: three in Subfamily 5 (F249, E410, L411 on *Vaccinium duclouxii* Vdu1) and two in Subfamily 4 (E249, G399 on the *Vaccinium duclouxii* SF4 representative). Subfamily 2 yielded no branch-site signal in the locus-deduplicated data (2ΔlnL = −0.08, Bonferroni-adjusted *p* = 1.0) and is interpreted as a case of relaxed selective constraint rather than positive selection. Structurally, the five candidate residues map to the C-terminal cytoplasmic regulatory regions of their respective reference proteins rather than to the transmembrane pore-lining helices as follows: the three SF5 sites were assigned to specific positions on the AlphaFold2 model of Vdu1 (Cα–Cα geometry and per-residue pLDDT reported), whereas the two SF4 sites were assigned to the C-terminal cytoplasmic domain of the SF4 reference protein based on DeepTMHMM topology. Together, these findings describe a pattern of compact retention coupled with regulatory-region sequence divergence, consistent with the hypothesis that the *Vaccinium* ALMT family has undergone lineage-specific sequence divergence at its cytoplasmic regulatory surfaces while preserving its essential ancestral channel architecture. The five identified sites define candidates for downstream functional validation; among them, the three SF5 sites (F249, E410, and *L411)* are immediately amenable to structure-guided mutagenesis based on the Vdu1 AlphaFold2 model, whereas the two SF4 sites (E249, G399) first require an independent structural model of the SF4 reference protein and complementary screening of sequence variation across *Vaccinium* germplasm to identify the most informative downstream experimental design.

## Figures and Tables

**Table 2:** Subfamily-specific *Ka/Ks* elevation in within-Vaccinium pairwise comparisons across the six major ALMT orthogroups, computed on the locus-deduplicated 145-locus dataset (per-OG raw KaKs_Calculator outputs in Supplementary Data S7). Pair counts equal C(n,2) per OG modulo pairs for which KaKs_Calculator returned NA. Fold-change is the ratio of median Vac–Vac to median Out–Out pairwise *Ka/Ks*; *p*-values are from one-tailed Mann–Whitney U tests (Vac–Vac > Out–Out) under the scipy.stats.mannwhitneyu asymptotic implementation, with Bonferroni correction across the six OG-level tests (multiplier × 6). ns, not significant (adjusted *p* > 0.05).

Orthogroup	Subfamily	*n*	*Median Ka/Ks (Out–Out)*	*Median Ka/Ks (Vac–Vac)*	Fold-Change	*p*-Value
OG0001431	1	38	0.159	0.159	1.00×	ns (raw *p* = 0.109; adj. *p* = 0.653)
OG0002701	2	31	0.234	0.358	1.53×	1.73 × 10^−3^ (adj. 1.04 × 10^−2^)
OG0001688	3	26	0.214	0.208	0.97×	ns (raw *p* = 0.462; adj. *p* = 1.000)
OG0006223	4	19	0.156	0.208	1.33×	8.60 × 10^−5^ (adj. 5.16 × 10^−4^)
OG0008930	5	16	0.270	0.508	1.88×	2.49 × 10^−4^ (adj. 1.49 × 10^−3^)
OG0012384	6	12	0.116	0.118	1.02×	ns (raw *p* = 0.640; adj. *p* = 1.000)

## Data Availability

All genome assemblies and gene annotations analyzed in this study are publicly available through NCBI RefSeq (https://www.ncbi.nlm.nih.gov/refseq/; accessed on 15 April 2026), the Genome Database for *Vaccinium* (GDV; https://www.vaccinium.org/; accessed on 15 April 2026), and the NGDC Genome Warehouse (GWH; https://ngdc.cncb.ac.cn/gwh; accessed on 15 April 2026); accession numbers and assembly versions are listed in Table S1. Custom analysis scripts, alignments, codon alignments, gene trees, orthogroup assignments, Ka/Ks tables, PAML branch-site outputs, MCScanX synteny blocks, and the AlphaFold2 model for the OG0008930 representative are deposited at https://github.com/ruiyifan7-boop/vaccinium_almt (accessed on 20 May 2026).

## Supplementary Materials

The following supporting information can be downloaded at https://www.mdpi.com/article/10.3390/plants15132086/s1. Table S1: Genome assemblies and accession numbers; Table S2: Final 145 non-redundant ALMT loci with per-gene validation columns (including TAIR locus IDs for Arabidopsis entries and DeepTMHMM TM topology); Table S3: Excluded alternative-transcript audit; Table S4: Vdu1 DeepTMHMM transmembrane topology; Table S5: Pairwise Ka/Ks values; Table S6: BEB positively selected sites; Table S7: Duplication-mode and synteny classification (updated for 24 locus-level MdALMTs); Supplementary Data S1–S7: Standard-format Word/PDF compilation of the following supporting datasets: Data S1, MAFFT and trimAl alignments of all 145 locus-level ALMTs; Data S2, IQ-TREE consensus and ML trees; Data S3, per-subfamily codon alignments; Data S4, PAML branch-site outputs; Data S5, Vdu1 AlphaFold2/ColabFold model information; Data S6, DeepTMHMM output for all 145 ALMT loci; and Data S7, per-OG raw Ka/Ks pair tables from KaKs_Calculator.
