# Supplementary material for: Compact Retention and Lineage-Specific Sequence Divergence of ALMT Genes in Acidophilic Vaccinium
Source: Plants (Basel). 2026 Jul 4;15(13):2086. doi: 10.3390/plants15132086 (PMC13364002; doi:10.3390/plants15132086)
Supplement: Supplementary file 1 [file plants-15-02086-s001.zip › Supplementary_Data_S1-S7.pdf]

Supplementary Materials

Compact Retention and Lineage-Specific Sequence Divergence of ALMT Genes in Acidophilic Vaccinium

Bin Li [1,2], Wenhan Cheng [1,2], Xianyang Zhao [1,2], Rui Chen [1,2], and Ruiyi Fan [1,2,*]

1 College of Food and Biology, Jingchu University of Technology, Jingmen 448000, China; libin0118@outlook.com (B.L.); kaven_53@163.com (W.C.); xyzhao2018@163.com (X.Z.); chenrui@jcut.edu.cn (R.C.)

2 Hubei Engineering Research Center for Specialty Flowers Biological Breeding, Jingmen 448000, China

* Correspondence: fanruiyi@outlook.com or fanruiyi@jcut.edu.cn

Supplementary Data Inventory

Data	Content	Main manuscript citation
S1	Family-wide MAFFT and trimAl alignments	Methods 2.3; Figure 2
S2	IQ-TREE consensus tree, ML tree, report, and run log	Methods 2.3; Figure 2
S3	Per-subfamily codon alignments for SF5, SF4, and SF2	Methods 2.6; Figure 4
S4	PAML branch-site output summary for SF5, SF4, and SF2	Methods 2.6; Figure 4
S5	Vdu1 AlphaFold2/ColabFold structural model summary	Methods 2.7; Figure 5
S6	DeepTMHMM topology output summary for 145 ALMT loci	Methods 2.7; Figure 5
S7	Per-OG Ka/Ks output summary	Methods 2.5; Table 2; Figure 3

Supplementary Data S1. MAFFT and trimAl alignments

Family-wide ALMT protein alignments before and after trimAl filtering.

File	Format	Records	Alignment length	Size
Supplementary_Data_S1a_ALMT_MAFFT_alignment_145.fasta	FASTA	145	1750	261.7 KB
Supplementary_Data_S1b_ALMT_trimAl_326col.fasta	FASTA	145	326	48.4 KB

Supplementary Data S2. IQ-TREE consensus and ML trees

Family-wide maximum-likelihood phylogenetic analysis of 145 ALMT loci.

Item	Value
Software	IQ-TREE 1.6.12 built Aug 15 2019
Best-fit model	JTT+F+R5
Log-likelihood	-25,667.5648 (s.e. 829.8829)
Bootstrap/SH-aLRT	1000 ultrafast bootstrap replicates and 1000 SH-aLRT replicates
Files included	Supplementary_Data_S2a_ALMT_iqtree_consensus_145.nwk; Supplementary_Data_S2b_ALMT_iqtree_ML_145.treefile; Supplementary_Data_S2c_ALMT_iqtree_log.iqtree; Supplementary_Data_S2d_ALMT_iqtree_runlog.log

Supplementary Data S3. Per-subfamily codon alignments

PHYLIP-format codon alignments used for the three PAML branch-site tests.

File	Subfamily	Orthogroup	Sequences	Codon length	Size
Supplementary_Data_S3a_SF5_OG0008930_codon_alignment.phy	SF5	OG0008930	16	1917	30.1 KB
Supplementary_Data_S3b_SF4_OG0006223_codon_alignment.phy	SF4	OG0006223	19	2010	37.4 KB
Supplementary_Data_S3c_SF2_OG0002701_codon_alignment.phy	SF2	OG0002701	31	1881	57.1 KB

Supplementary Data S4. PAML branch-site outputs

Branch-site Model A and null Model A outputs for the three orthogroups tested with terminal Vaccinium branches as foreground.

Subfamily	Orthogroup	lnL Model A	lnL null	2ΔlnL	Raw p	Adjusted p	BEB sites	Interpretation
SF5	OG0008930	−9406.967418	−9427.293969	40.65	9.1×10^{-11}	2.7×10^{-10}	F249, E410, L411	Positive-selection signal detected
SF4	OG0006223	−14997.417902	−15003.892317	12.95	$\sim 1.6 \times 10^{-4}$	$\sim 4.8 \times 10^{-4}$	E249, G399	Positive-selection signal detected
SF2	OG0002701	−27133.311465	−27133.269342	−0.08	0.5	1.0	None	Relaxed constraint

Supplementary Data S5. Vdu1 AlphaFold2/ColabFold structural model summary

Summary of the Vdu1 Subfamily 5 structural model used for mapping BEB-significant residues.

Metric	Value
Source file	Supplementary_Data_S5_Vduc1_Subfamily5_AlphaFold2_model.pdb
Model length	420 residues
Mean pLDDT	80.49
Median pLDDT	87.91
pLDDT range	26.12–97.38

BEB-significant residue mapping:

Mapped site	PDB residue name	pLDDT	Note
F249	PHE	78.06	BEB-significant SF5 candidate site
E410	GLU	92.62	BEB-significant SF5 candidate site
L411	LEU	93.44	BEB-significant SF5 candidate site

Supplementary Data S6. DeepTMHMM output for all ALMT loci

Summary of transmembrane topology predictions for the 145 retained ALMT proteins.

Predicted TM helices	Number of proteins
4	4
6	137
7	2
8	2

Output files:

File	Description	Size
per_protein_tm.tsv	Per-protein length and TM count	2.2 KB
predicted_topologies.3line	Three-line topology predictions	150.6 KB
TMRs.gff3	GFF3-formatted topology coordinates	59.9 KB

Supplementary Data S7. Per-OG raw Ka/Ks pair tables

Summary of per-orthogroup pairwise Ka/Ks outputs used for Table 2 and Figure 3.

OG	Subfamily	n Vac–Vac	n Out–Out	Median Out–Out	Median Vac–Vac	Fold	Adjusted p	Interpretation
OG0001431	SF1	105	211	0.1587	0.1590	1.00×	0.6531	Not significant after correction
OG0002701	SF2	6	344	0.2338	0.3580	1.53×	0.01036	Bonferroni-significant elevation
OG0001688	SF3	55	103	0.2139	0.2079	0.97×	1	Not significant after correction
OG0006223	SF4	35	39	0.1561	0.2079	1.33×	0.0005162	Bonferroni-significant elevation
OG0008930	SF5	10	55	0.2695	0.5075	1.88×	0.001494	Bonferroni-significant elevation
OG0012384	SF6	10	21	0.1155	0.1175	1.02×	1	Not significant after correction

Per-orthogroup output files:

File set	Description
kaks_summary.tsv	Summary statistics for the six major ALMT orthogroups
OG0001431.kaks; OG0001688.kaks; OG0002701.kaks; OG0006223.kaks; OG0008930.kaks; OG0012384.kaks	Per-orthogroup pairwise Ka/Ks tables generated by KaKs_Calculator
